# Assessing the Relationship between the Baseline Value of a Continuous Variable and Subsequent Change Over Time

**DOI:** 10.3389/fpubh.2013.00029

**Published:** 2013-08-23

**Authors:** Arnaud Chiolero, Gilles Paradis, Benjamin Rich, James A. Hanley

**Affiliations:** ^1^University Hospital Center, Institute of Social and Preventive Medicine (IUMSP), University of Lausanne, Lausanne, Switzerland; ^2^Department of Epidemiology, Biostatistics, and Occupational Health, McGill University, Montreal, QC, Canada; ^3^McGill University Health Center Research Institute, Montreal, QC, Canada; ^4^Public Health Institute of Quebec, Montreal, QC, Canada

**Keywords:** baseline value, change, measurement error, regression to the mean, mathematical coupling

## Abstract

Analyzing the relationship between the baseline value and subsequent change of a continuous variable is a frequent matter of inquiry in cohort studies. These analyses are surprisingly complex, particularly if only two waves of data are available. It is unclear for non-biostatisticians where the complexity of this analysis lies and which statistical method is adequate. With the help of simulated longitudinal data of body mass index in children, we review statistical methods for the analysis of the association between the baseline value and subsequent change, assuming linear growth with time. Key issues in such analyses are mathematical coupling, measurement error, variability of change between individuals, and regression to the mean. Ideally, it is better to rely on multiple repeated measurements at different times and a linear random effects model is a standard approach if more than two waves of data are available. If only two waves of data are available, our simulations show that Blomqvist’s method – which consists in adjusting for measurement error variance the estimated regression coefficient of observed change on baseline value – provides accurate estimates. The adequacy of the methods to assess the relationship between the baseline value and subsequent change depends on the number of data waves, the availability of information on measurement error, and the variability of change between individuals.

## Introduction

Analyzing the relationship between the baseline value and subsequent change of a continuous variable is a frequent matter of inquiry in cohort studies. For instance, researchers may want to estimate what is the average expected body mass index (BMI) change in children considering their initial BMI: if BMI is elevated, will the subsequent change in BMI be larger or smaller than if BMI is initially low? Is there a differential baseline effect on change (1–3)? Baseline BMI is typically positively associated with subsequent change in BMI in children from age 5 and above (4). In the study of such relationship, the fundamental goal is to estimate the association between the *true* initial value of the variable of interest and *true* subsequent change (5, 6). However, the observed values may not correspond to the true (unobservable) values and observed change may not correspond to true change. How can the relationship be properly assessed with minimum bias using observed values?

It is important here to distinguish two questions: one is how to estimate to which extent change is related to the initial value; the other is how to estimate change in a variable of interest in relation to other variables, given the initial value of the variable of interest. The latter question has been extensively addressed in previous reviews (7–10). In this tutorial style paper, our aim is to address the former question. Previous reviews on this topic were quite technical and sometimes highly conflicting in their conclusions (1, 2, 11–16). As a result, it remains often unclear for non-biostatisticians where the complexity of this analysis lies and which statistical method is adequate to properly address this question, especially when only two waves of data are available. Therefore, with the help of simulated longitudinal data of BMI in children, we review methods for the analysis of the association between baseline value and change of a continuous variable in cohort studies.

## The Problem

Let’s suppose a cohort of subjects for whom the continuous random variable *Y* has been measured twice during follow-up. *Y*_1_ and *Y*_2_ are the true values of *Y* at initial and follow-up time, respectively. Due to within-subject short-term biological variability and imperfect measurement methods, there is some measurement error in the estimate of *Y*_1_ and *Y*_2_ (see Glossary). Hence, *U*_1_ and *U*_2_ are the observed value of *Y*_1_ and *Y*_2_: *U*_1_ = *Y*_1_ + *E*_1_ and *U*_2_ = *Y*_2_ + *E*_2_, where *E*_1_ and *E*_2_ are independent random measurement errors. The observed change is *U*_2_ − *U*_1_.

It appears trivial to estimate the association between the baseline value and subsequent change, e.g., by the regression of *U*_2_ − *U*_1_ on *U*_1_ or by the correlation between *U*_2_ − *U*_1_ and *U*_1_. However, these methods result in a negatively biased estimate (1, 11–16). For instance, a strong and inverse association between the initial BP level and subsequent BP change was reported in many trials of antihypertensive drugs, leading to conclusion that drugs were more potent among patients with very high level of BP (15). The inverse association disappeared when apparently appropriate statistical analyses were used (15).

Assessing the genuine association between baseline value and subsequent change of a continuous variable is complex, especially if only two waves of data are available. Mathematical coupling, measurement error, variability of change between individuals, and regression to the mean are interrelated concepts which need to be addressed to better understand issues at stake in such analyses (see Glossary) (17–22).

The observed correlation between the initial value (*U*_1_) and change (*U*_2_ − *U*_1_) results in part from the mathematical coupling between the two terms (2, 23–25). Mathematical coupling occurs when one variable is part of another (24, 25). It is a common problem in physiology where correlations are assessed between variables that are calculated using a common set of measured variables (22, 24). The calculated variables share a common source of variation which introduces a relation between the variables that has no physiological basis (22, 23). A separated problem is that any error in the measurement of the shared component creates a coupling of errors between the coupled variables (23).

In our case, *U*_1_ and *U*_2_ − *U*_1_ share the common variable *U*_1_. If we suppose that *U*_1_ and *U*_2_ are two independent random variables with identical variance, it can be shown that the observed correlation between *U*_1_ and *U*_2_ − *U*_1_ is −1/√2 (13) (for the derivation, see Appendix). In this case, *Y*_1_ and *Y*_2_ are not correlated. In general and in the simulations below, *Y*_1_ and *Y*_2_ are not independent variables and are correlated. The correlation is often positive, between 0 and 1, but rarely attains 1 because perfect correlation would only occur if every individual underwent exactly the same (true) change, which is not the case in general (2). Indeed, there is some variability of true change between individuals. Regression to the mean is the expression of imperfect correlation between repeated measures (19–21) (see Glossary). The lower the correlation between *Y*_1_ and *Y*_2_, the larger is the effect of mathematical coupling (25), the greater is the amount of regression to the mean, and the larger is the impact on the correlation between *U*_1_ and *U*_2_ − *U*_1_. Error in the measurement of *Y*_1_ and *Y*_2_ is also associated with a low correlation between *U*_1_ and *U*_2_ and has an impact on the correlation between *U*_1_ and *U*_2_ − *U*_1_.

## How to Assess the Association between the Baseline Value and Subsequent Change?

If more than two measures per individual are available, the linear random effects model or individual growth curve modeling (see Glossary) is a standard method to assess the relationship between change in a continuous variable and its correlates, including the baseline level (28, 35, 36).

**Figure 1:**
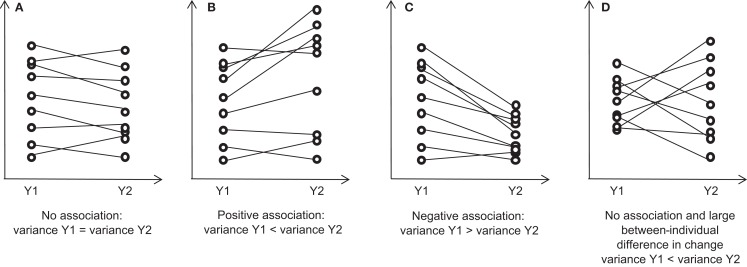
**Each panel shows the spread (to illustrate the variance) of a continuous variable *Y* measured at initial (*Y*_1_) and follow-up (*Y*_2_) times under various assumptions**. “True” values are shown (without measurement error). **(A)** If there is no association between *Y*_1_ and subsequent change (*Y*_2_ − *Y*_1_), the spread may remain constant during follow-up; **(B)** If there is a positive association, the spread may increase during follow-up; **(C)** If there is a negative association, the spread may decrease during follow-up; **(D)** If there is no association and a large between-individual difference in change, the spread may increase during follow-up.

Linear random effects model cannot be used with two waves of data. In such a case, the pattern of changes in the spread of the data during follow-up may help to reveal the existence of an association between the baseline value and subsequent change (1, 13, 26, 27). The variance may not change if there is no association between baseline and change (Figure 1A). If there is an association, the variance may either increase (positive association, increasing spread of the data) or decrease (negative association, decreasing spread of the data) (Figures 1B,C). For example, the increasing spread of BMI during childhood may suggest that having a high BMI early in childhood is associated with a larger BMI gain in the following years (4, 28). The pattern of individual time paths showing positive correlation between initial value and subsequent change is called fan spread (29). A variance ratio test exists to test for the equivalence of variance between two correlated variables (30). While comparing variance is intuitively appealing, it is misleading in many situations. For instance, if there are large between-individual differences in change of the variable of interest during follow-up, variance can increase even if there is no association between baseline and subsequent change (Figure 1D) (29).

Oldham proposed more than 50 years ago assessing the association between *U*_2_ − *U*_1_ and the average (*U*_1_ + *U*_2_)/2, rather than between *U*_2_ − *U*_1_ and *U*_1_ (31, 32). Due to random error, the initial measure *U*_1_ is merely an imperfect surrogate for an unobservable true baseline level *Y*_1_ and the average may be a better indicator of the true level of *Y*. Furthermore, the difference in the random errors of *U*_1_ and *U*_2_ is uncorrelated with their average. Nevertheless, this method allows the unbiased estimation of a different statistic, that is, of the correlation between change and the value at the mid-time point between *Y*1 and *Y*2, but not of the correlation between change and the value at baseline.

To avoid correlated errors due the mathematical coupling between *U*_1_ and *U*_2_ − *U*_1_, one may assess the association between *U*_2_ − *U*_1_ and another measure obtained relatively close in time to *U*_1_ (16), e.g., a repeated measure *U*_1bis_. While this method avoids some bias inherent to the estimate obtained by the crude association, the estimate tends to be biased toward the null due to the measurement error on *U*_1bis_, i.e., a regression dilution bias (see Glossary) (33).

Blomqvist proposed another method which consists in adjusting the estimated regression coefficient of change on baseline value accounting directly for measurement error (5, 6, 34). This method requires accurate information on the variance of measurement error: the ratio of measurement error variance to total variance is used to make a correction of the crude estimate of the regression or correlation coefficient [adjusted regression coefficient = (crude regression coefficient + *k*)/(1 − *k*), with *k* = variance of measurement error/total variance; see also below].

These methods listed in Table 1 can result in different estimates of the association between baseline value and subsequent change, and they cannot be used in all contexts. To assess the strengths and weaknesses of these methods, we applied each of them to examine the association between baseline value and subsequent change of BMI to simulated data of a cohort of children.

**Table 1:** **Methods to assess the association between the baseline value and subsequent changes of a continuous variable**.

Method	Description	Comment
Crude method, e.g.	E.g., simple correlation between *U*_2_ − *U*_1_ and *U*_2_	Used with one initial and one follow-up measure.
Comparison of variances (13)	Analysis of the change in variance during follow-up	Used with one initial and one follow-up measure.
Oldham’s method (31)	Assessment of the relationship between *U*_2_ − *U*_1_ and (*U*_2_ + *U*_1_)/2	Used with one initial and one follow-up measure.
Repeated measurement method (16)	Assessment of the relationship between *U*_2_ − *U*_1_ and *U*_1bis_, a measure obtained relatively close in time to *U*_1_	If one initial and one follow-up measure are available, one additional measure close to the initial one is needed.
Blomqvist’s method (5, 6, 32, 34)	With an adjustment accounting directly for measurement error	If one initial and one follow-up measure are available, quantitative information on measurement error is needed.
Linear random effects regression modeling (LREM) (1, 30, 33)	Section “Glossary”	If one initial and one follow-up measure are available, at least one additional measure is needed.

## Simulations

### Scenarios

We simulated a cohort study of 500 children randomly selected from the general population who underwent BMI measurement each year between the ages of 5 and 10. We assumed that each child’s true BMI followed an individual linear growth with time with a specific initial true BMI (intercept) at the age of 5 and a specific rate of change (slope) between the ages of 5 and 10. At the population level, intercept and slope are random variables. Their distribution reflects between-individual variability in the true initial BMI and true change.

First, we simulated data following a “realistic” scenario, i.e., with BMI changes throughout time close to what would be observed in real life (4, 28). At the initial time (age 5), the mean true BMI was 15.0 kg/m^2^ (SD: 1.2) (normal distribution). True BMI changed at a mean rate of +0.4 kg/m^2^ per year between the ages of 5 and 10. The true BMI change varied between children (SD of yearly change: 0.2) and was positively associated with baseline level (correlation: +0.6). Using these parameters in a linear random effects regression model (Glossary), we simulated true BMI values at ages 5, 6, 7, 8, 9, and 10. Observed BMI values were computed by adding a relatively small random measurement error (SD of error: 0.3) to the true BMI values. Because error occurs at random, the means of errors are zero and the means of observed values are close to the mean of true values. Second, we simulated data along alternative scenarios, including a larger measurement error (SD: 1.2) and a larger between-individual variability in true yearly change (SD of yearly change: 0.4). Each simulation was repeated 1000 times.

### Analyses

We first compared changes in the spread (SD) of measured BMI at the beginning of follow-up at age 5 (initial value: *U*_1_) and at the end of the follow-up at age 10 (*U*_2_) for each scenario. Second, we estimated the association between baseline (*Y*_1_) and change (*Y*_2_ − *Y*_1_) using the methods described in Table 1 including:

(1) Linear random effects modeling method (29, 35): we fitted a linear random effects regression model of BMI on age using all available data, i.e., measured BMI at ages 5, 6, 7, 8, 9, and 10. We assessed the correlation *r*_random_ between the random coefficient of the slope and the random coefficient of the intercept;
(2) Crude method: we assessed the correlation *r*_crude_ between *U*_2_ − *U*_1_ (at initial time and at age 10) and *U*_1_ (at initial time);
(3) Oldham’s method (31): we assessed the correlation *r*_Oldham_ between *U*_2_ − *U*_1_ (at initial time and at age 10) and (*U*_1_ + *U*_2_)/2;
(4) Repeated measurement method (16): we assessed the correlation *r*_repeated_ between *U*_2_ − *U*_1_ (at initial time and at age 10) and *U*_1bis_ (at initial time), which is a simulated repeated measurement of *Y* at the initial time;
(5) Blomqvist’s method (32, 34): first, we assessed the regression coefficient *b*_crude_ of *U*_2_ − *U*_1_ (at initial time and at age 10) on *U*_1_ (at initial time). Second, we computed the regression coefficient *b*_Blomqvist_ adjusted for measurement error as *b*_Blomqvist_ = (*b*_crude_ + *k*)/(1−*k*), with *k* = variance of measurement error/total variance = 1 – intra-class correlation (ICC) between (short-term) repeated measurements (Glossary). The corresponding correlation *r*_Blomqvist_ was estimated. We compared estimates assuming that the information on the variance of measurement error (to compute *k*) was accurate, underestimated, or overestimated.

Simulations and analyses were conducted using the statistical package R 2.10.1 (R Project for Statistical Computing; http://www.r-project.org/).

### Results of the simulations

Assuming a relatively low measurement error and small between individual variability of change, the observed mean BMI and standard deviation (SD) of a simulated cohort of 500 children are shown as an illustration in Table 2. Mean BMI increased linearly with age during that period. The SD also increased indicating an increase in the variance. Such a data pattern is typically observed if there is a positive correlation between initial value and subsequent change and is named fan spread (29).

**Table 2:** **Measured body mass index (BMI) and standard deviation (SD) of the 500 children followed-up annually between the ages of 5 and 10**.

Age (years)	BMI (kg/m^2^)	SD (kg/m^2^)
5	15.0	1.2
6	15.4	1.3
7	15.8	1.5
8	16.2	1.6
9	16.6	1.8
10	17.0	1.9

**Figure 2:**
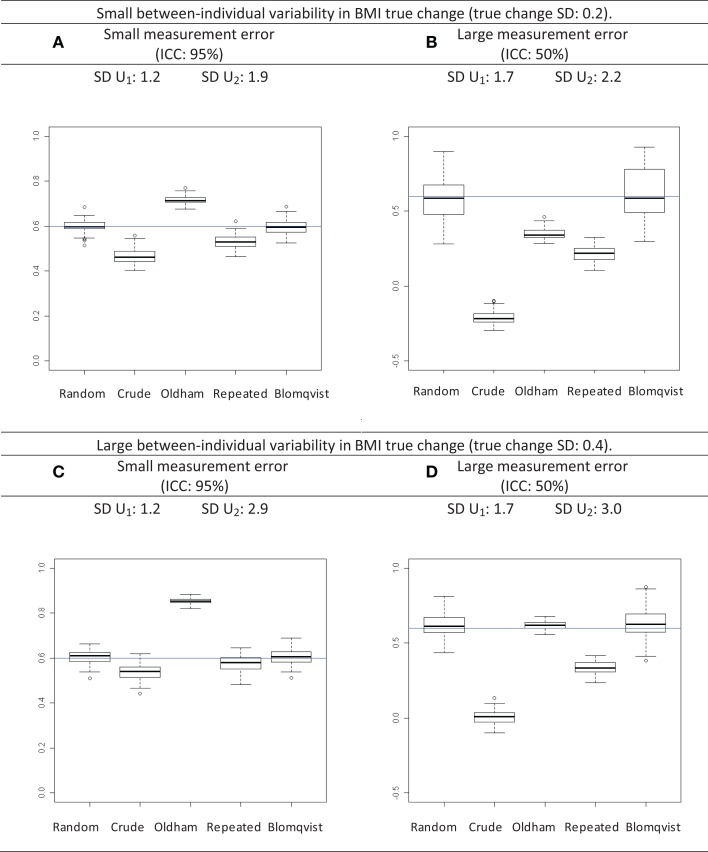
**Distribution of the estimates of correlation coefficients between change and initial value following different scenarios**. The standard deviation (SD) of measured BMI at age 5 (initial time; *U*_1_) and at age 10 (follow-up time; *U*_2_) are reported. The horizontal line is the expected correlation. The box plot shows the mean and the 25th and 75th percentile, and the whiskers extend to 1.5 times the interquartile range from the box. Random: estimate using a linear random effects model; Crude: crude correlation between initial value and change; Oldham: estimates using Oldham’s method; Repeated: estimates using repeated measurement method; Blomqvist: estimates using Blomqvist’s method. **(A)** Results in case of small between-individual variability in BMI true change and of small measurement error. **(B)** Results in case of small between-individual variability in BMI true change and of large measurement error. **(C)** Results in case of large between-individual variability in BMI true change and of small measurement error. **(D)** Results in case of large between-individual variability in BMI true change and of large measurement error.

Estimates of the correlation between the baseline and change are shown in the Figures 2A,B assuming a relatively small variability in BMI change between individuals (true yearly change SD: 0.2) and Figures 2C,D assuming a relatively large variability in BMI change between individuals (true yearly change SD: 0.4), respectively.

#### Relatively small change variability between individuals

Assuming a relatively low measurement error variance (ICC of 95%) (Figure 2A), the average estimate based on the linear random effects regression model *r*_random_ was +0.6 as expected. The crude estimate *r*_crude_ was slightly negatively biased, *r*_Oldham_ was slightly positively biased, and *r*_repeated_ was slightly negatively biased. The estimated correlation *r*_Blomqvist_ was on average equal to the expected correlation *r*_random_. SD increased from 1.2 to 1.9 kg/m^2^ during follow-up. If the measurement error variance was relatively large (ICC: 50%) (Figure 2B), *r*_crude_ and *r*_repeated_ were more negatively biased than if measurement error was small, *r*_Oldham_ was negatively biased and closer to the null value. *r*_Blomqvist_ was on average equal to the expected correlation. The SD increase was less important than in (Figure 2A).

#### Relatively large variability in change between individuals

Similar patterns for *r*_crude_, *r*_repeated_, and *r*_Blomqvist_ were observed for large compared to small change variability between individuals. However, *r*_Oldham_ was more biased when measurement error variance was small (Figure 2C) and less biased when measurement error variance was large (Figure 2D). SD increased systematically and the amount of SD increase during follow-up was much larger in most cases with large than with small change variability between individuals (Figures 2C,D).

#### Blomqvist’s method with variance of measurement error under or overestimated

If the variance of measurement error was underestimated, *r*_Blomqvist_ was negatively biased (data not shown). Conversely, if the variance of measurement error was overestimated, *r*_Blomqvist_ was biased away from the null: the correlation was overestimated if the true correlation was positive and underestimated if the true correlation was negative.

## Discussion

Different methods exist to assess the association between the baseline and subsequent change in the value of a continuous variable (Table 1). Our simulation study indicates that the crude correlation was systematically negatively biased. In some cases, analyzing the pattern of change in the spread of data helped identify the existence of an association. In most scenarios, Oldham’s method did not allow estimating the correlation. Using the repeated measurement method, the correlation was biased toward the null. Using Blomqvist’s method, the estimated correlation was on average equal to the true correlation, assuming an accurate estimate of the measurement error variance. As expected, the estimated correlation using linear random effects modeling method was equal to the true correlation.

The linear random effects model (or individual growth curve modeling) has received common acceptance to analyze change in a continuous variable and its correlates, including the baseline level (1, 28, 30, 33). However, this method requires multiple measurements and cannot be used if only two waves of data are available. Some of the other listed methods (Table 1) can be used with two waves of data, but with caution. In fact, assumptions have to be made either on the amount of measurement error or on the amount of between individuals variability of (true) change to properly interpret estimates with any of these methods, and it may be difficult to check the validity of these assumptions. For instance, analyzing the variance offers insights only if there is *a priori* a small variability of change between individual. Blomqvist’s method provides accurate estimates and this method is in fact an application of the linear random effects model [see Edland (32) for an in depth explanation]. However, Blomqvist’s method requires having an accurate estimate of the variance of measurement error, based on short-term repeated measurements or from external sources (e.g., previous studies). Some authors have argued that Blomqvist’s method does not correct for regression to the mean caused by between-individual variability of change (2) but our simulation indicates that this method provides accurate estimate of the relation between initial value and change assuming different levels of variability of change between individuals.

The estimate of the correlation was biased in many cases with Oldham’s method. While this method has been used extensively (15, 31), it has been criticized notably because it is difficult to understand why we should assess the relation between (*U*_1_ + *U*_2_)/2 and *U*_2_ − *U*_1_ if the aim is to estimate the relation between *Y*_1_ and *Y*_2_ − *Y*_1_. Indeed, this method allows the unbiased estimation of a different statistic, that is, of the correlation between change and the value at the mid-time point between *Y*1 and *Y*2, but not of the correlation between change and the value at baseline. Repeated measurement method may be satisfactory if measurement error is small: in that case, the estimate is slightly biased toward the null, due to the regression dilution bias (33). However, measurement errors of *U*_1_ and *U*_1bis_ (a repeated measurement of *Y* at the initial time) may be correlated. If this is the case, the association between *U*_2_ − *U*_1_ and *U*_1bis_ will still be affected by regression to the mean and will be negatively biased. A correlation between errors is difficult to ascertain but will be likely if the initial value (*U*_1_) and the repeated measure (*U*_1bis_) are gathered close in time. Furthermore, since a third measure is required to use repeated measurement method, random effect modeling should be preferred in this circumstance.

The linear random effects method and Blomqvist’s method account for random intra-individual variability. Intra-individual variability has two components, one due to imperfect measurement methods and another due to the (short-term) biological variability (33) but, in practice, one component cannot most often be separated from the other. Measurement error is typically assessed by the variability of repeated measurements over a short period of time. Such a method may be insufficient to capture the whole biological variability. For example, assessing BP a few times at one visit may not capture the full random individual variability of BP (37, 38). Consequently, with limited numbers of measurements or limited information on the variance of measurement error, the linear random effects method and Blomqvist’s method may not capture the genuine association between baseline value and subsequent change of a continuous variable.

We simulated a cohort of 500 subjects examined at the same ages to simplify our illustration. Linear random effects model as well as Blomqvist’s method can be used with data collected at different spacing (in our case different ages) (32, 35). Random variability in the estimates would have been greater for all methods with a smaller cohort size, potentially blurring the difference between the methods. No confounding factor was taken into account in the simulations. Linear random effects model can directly take account of confounding factors. Furthermore, we assumed in our simulation a linear growth with time. While this may be approximately true over short periods of time, such a model is not realistic over a long interval for most biologic variables. More complex non-linear patterns of growth over time can be analyzed by random effects models if enough measurements are available (32, 35). However, in case of non-linear growth, the question of whether change depends on baseline value or not becomes highly complex. If only two waves of data are available and a linear approximation of growth is not realistic, there is no satisfactory method. Other methods have been proposed to assess the association between the initial value and subsequent change, e.g., generalization of Blomqvist’s method by Edland (32) or structural regression and multilevel modeling (13, 39).

Finally, it is important to underscore that the assessment of the true association between the baseline value and subsequent change (or between level and slope) is easily misinterpreted. At best, such assessment helps predict the expected future change of a variable given previous (true) level. However, while it is tempting, no simple etiological inference can be made in most cases (32). When modeling progressive conditions (such as elevated BMI), it is important to consider that differential progression prior to the initial measurement can induce an association between level at any time and change (32). Actually, the association between initial level and subsequent change can be explained by changes prior to the initial measurement, a so-called “horse racing effect” in case of positive association between level and change (4, 40), and the strength of the association will depend on the timing at which initial and follow-up values were measured (1).

## Summary

To assess the genuine association between the baseline value of a continuous variable and subsequent change, assuming a linear growth with time:

- In general, it is better to rely on multiple measurements at different times to account for the variability of changes between individual and for measurement error.
- If only two waves of data and accurate estimation of the variance of measurement error are available, our simulation shows that Blomqvist’s method provides accurate estimation.
- If more than two waves of data are available, a linear random effects model provides accurate estimation.
- In all cases, researchers should be cautious on causal interpretations of the relationship between baseline value and subsequent change.

## Glossary

**Measurement error:** Short-term within-subject variability. It has two components: (1) an error due to the measurement method itself and (2) a short-term biological variability. The error occurs at random if it fluctuates in an unpredictable manner around the true value (which is estimated by the mean of repeated measurements) (33).

**Mathematical coupling:** Because the initial value (*Y*_1_) and the change from initial value (*Y*_2_ − *Y*_1_) share a common variable, there is a mathematical coupling between *Y*_1_ and *Y*_2_ − *Y*_1_ (2, 23–25). This coupling induces a correlation between *Y*_1_ and *Y*_2_ − *Y*_1_.

**Intra-class correlation (ICC):** ICC is the proportion of variability due to the between-subject variability. It is the ratio of between-subject variance to total variance, the latter being the sum of between-subject variance and (short-term) within-subject variance. The within-subject variance is the measurement error variance and can be estimated in longitudinal studies by repeated measurements over a short period of time. ICC is also called the reliability coefficient.

**Regression to the mean (RTM):** RTM (also called regression effect, regression paradox, or regression fallacy) affects any variable measured with some random error. It is the expression of imperfect correlation between repeated measures and results from some selection process. For example, if the initial measure *U*_1_ is elevated, the probability of having a lower second measure *U*_2_ is – strictly by chance – greater than having a higher measure. The reverse is true if *U*_1_ is initially low: the probability of having a higher follow-up measure *U*_2_ is greater than having a lower measure. Whatever the initial measure, the following one is – on average- closer to the mean of multiple measures: it has regressed to the mean. The further away is the initial measure from the mean, the greater – on average – is the amount of RTM (19–21). RTM is what produces the correlation between initial value and subsequent change (1). RTM also manifests at group level when subjects are sampled based on their extremeness, i.e., above or below a given threshold, or due to random sampling variability. Due to RTM, the change observed in this group cannot be used as an estimate of the average change in the population.

**Regression dilution bias:** Occurs in the presence of random measurement error in the exposure: it biases toward the null the estimate of the relationship between the exposure (in our case, the initial value) and the outcome (in our case, the change) (33).

**Linear random effects regression model (LREM):** Used to model repeated longitudinal measures of a given variable *Y* throughout time, also called individual growth modeling (35). *Y* is assumed to follow a linear trajectory over time (or age) which differs from one individual to the other; *Y* is thus modeled for an individual *i* at time*_ij_* as *E*(*y_ij_*) = β_0_ + b_0_*_i_* + β_1_ × time*_ij_* + b_1_*_i_* × time*_ij_* where b_0_*_i_* and b_1_*_i_* are random coefficients for the intercept β_0_ and the slope β_1_, respectively. The regression of b_1_*_i_* on b_0_*_i_* or the correlation between b_1_*_i_* and b_0_*_i_* are indicators of the relationship between change in the level of a variable and its initial value (32, 35). Two-wave longitudinal data cannot be used to fit such model; additional measures are required.

### Appendix

The correlation *r* between *U*_1_ and *U*_2_ − *U*_1_ can be written as:

$$\begin{array}{llll} \gamma_{U2-U1,U1} & =\frac{\mathrm{cov}\left(U1,U2-U1\right)}{\sqrt[{2}]{\mathrm{Var}\left(U1\right)\times \mathrm{Var}\left(U2-U1\right)}},\; & & \;\\ & \frac{\mathrm{cov}\left(Y1+E1,Y2+E2-Y1-E1\right)}{\sqrt[{2}]{\mathrm{Var}\left(Y1+E1\right)\times \mathrm{Var}\left(Y2+E2-Y1-E1\right)}}.\; & & \; \end{array}$$

If we assume that that *E*1 and *E*2 are random variables with identical variance and that the covariance (Y_1_,Y_2_) is null (no correlation between *Y*_1_ and *Y*_2_), the equation can be simplified as

$$\begin{array}{c} \gamma_{U2-U1,U1}=\frac{\mathrm{cov}(Y1,\;Y2)-\text{Var}(Y)-\text{Var(}E\text{)}}{\sqrt[{2}]{\begin{array}{l} (\text{Var(}Y\text{)}+\text{Var(}E\text{))}\times 2\times [\text{Var}(Y)+\text{Var(}E\text{)}\\ \;-\text{Con(}Y1-Y1)] \end{array}}},\\ =\frac{-1}{\sqrt[{2}]{2}}=-0.71. \end{array}$$

## Authors’ Contributions

Arnaud Chiolero and James A. Hanley had the original idea for this work, which was developed with Gilles Paradis and Benjamin Rich. Arnaud Chiolero drafted the manuscript. Arnaud Chiolero and Benjamin Rich carried out the simulation under the supervision of James A. Hanley. Gilles Paradis, Benjamin Rich, and James A. Hanley revised the manuscript. All authors read and approved the final manuscript.

## Conflict of Interest Statement

The authors declare that the research was conducted in the absence of any commercial or financial relationships that could be construed as a potential conflict of interest.
